# Population Structure of *Sclerotinia subarctica* and *Sclerotinia sclerotiorum* in England, Scotland and Norway

**DOI:** 10.3389/fmicb.2017.00490

**Published:** 2017-04-04

**Authors:** John P. Clarkson, Rachel J. Warmington, Peter G. Walley, Matthew Denton-Giles, Martin J. Barbetti, Guro Brodal, Berit Nordskog

**Affiliations:** ^1^Warwick Crop Centre, School of Life Sciences, University WarwickWarwick, UK; ^2^Eden ProjectBodelva, UK; ^3^Institute of Integrative Biology, University of LiverpoolLiverpool, UK; ^4^Centre for Crop and Disease Management, Curtin UniversityBentley, WA, Australia; ^5^Faculty of Science, School of Agriculture and Environment, University of Western AustraliaCrawley, WA, Australia; ^6^Division of Biotechnology and Plant Health, Norwegian Institute of Bioeconomy ResearchÅs, Norway

**Keywords:** sclerotinia, sclerotiorum, subarctica, population, diversity, microsatellites, intergenic spacer region

## Abstract

*Sclerotinia* species are important fungal pathogens of a wide range of crops and wild host plants. While the biology and population structure of *Sclerotinia sclerotiorum* has been well-studied, little information is available for the related species *S. subarctica*. In this study, *Sclerotinia* isolates were collected from different crop plants and the wild host *Ranuculus ficaria* (meadow buttercup) in England, Scotland, and Norway to determine the incidence of *Sclerotinia subarctica* and examine the population structure of this pathogen for the first time. Incidence was very low in England, comprising only 4.3% of isolates while moderate and high incidence of *S. subarctica* was identified in Scotland and Norway, comprising 18.3 and 48.0% of isolates respectively. Characterization with eight microsatellite markers identified 75 haplotypes within a total of 157 isolates over the three countries with a few haplotypes in Scotland and Norway sampled at a higher frequency than the rest across multiple locations and host plants. In total, eight microsatellite haplotypes were shared between Scotland and Norway while none were shared with England. Bayesian and principal component analyses revealed common ancestry and clustering of Scottish and Norwegian *S. subarctica* isolates while English isolates were assigned to a separate population cluster and exhibited low diversity indicative of isolation. Population structure was also examined for *S. sclerotiorum* isolates from England, Scotland, Norway, and Australia using microsatellite data, including some from a previous study in England. In total, 484 haplotypes were identified within 800 *S. sclerotiorum* isolates with just 15 shared between England and Scotland and none shared between any other countries. Bayesian and principal component analyses revealed a common ancestry and clustering of the English and Scottish isolates while Norwegian and Australian isolates were assigned to separate clusters. Furthermore, sequencing part of the intergenic spacer (IGS) region of the rRNA gene resulted in 26 IGS haplotypes within 870 *S. sclerotiorum* isolates, nine of which had not been previously identified and two of which were also widely distributed across different countries. *S. subarctica* therefore has a multiclonal population structure similar to *S. sclerotiorum*, but has a different ancestry and distribution across England, Scotland, and Norway.

## Introduction

*Sclerotinia* species are important pathogens of a wide range of crop plants as well as many wild hosts. Of these, *S. sclerotiorum* (Lib.) de Bary is probably the best studied with a worldwide distribution and a wide host range of more than 400 plants including many important dicotyledonous crops and wild species (Boland and Hall, 1994). Some of the major crops affected include oilseed rape, soybean, sunflower, lettuce, carrot, potatoes, beans, and peas (Bolton et al., 2006). Infection of the majority of host plants is by ascospores released from apothecia produced through carpogenic germination of soilborne sclerotia, although direct infection by myceliogenic germination can occasionally occur (Hao et al., 2003). Apothecia are formed through sexual reproduction, and as *S. sclerotiorum* is predominantly homothallic, a multiclonal population structure has generally been observed in studies carried out on a variety of crop plants in Alaska, Australia, Brazil Canada, China, Iran, New Zealand, Turkey, UK, and USA using DNA fingerprinting (Kohn et al., 1991; Kohn, 1995; Cubeta et al., 1997; Carbone et al., 1999; Carpenter et al., 1999; Carbone and Kohn, 2001b; Hambleton et al., 2002; Phillips et al., 2002) or microsatellite genotyping (Sexton and Howlett, 2004; Sexton et al., 2006; Winton et al., 2006; Mert-Turk et al., 2007; Hemmati et al., 2009; Gomes et al., 2011; Attanayake et al., 2013; Clarkson et al., 2013; Aldrich-Wolfe et al., 2015; Lehner et al., 2015). In these studies, the typical population structure is such that one or a small number of clones is sampled at high frequency, with the remainder sampled only once or a few times (Kohn, 1995). The high frequency *S. sclerotiorum* clones found at a local scale can sometimes be sampled repeatedly over several years in the same locality and in some cases over a wider geographic area (Hambleton et al., 2002; Clarkson et al., 2013). There is, however, a limit to the geographic distribution of *S. sclerotiorum* clones; for instance, none of the *S. sclerotiorum* clones from oilseed rape and soybean identified by DNA fingerprinting in Canada (Kohn et al., 1991; Kohli et al., 1992, 1995; Hambleton et al., 2002) were found in various crops from different locations in the USA (Cubeta et al., 1997; Malvárez et al., 2007). The distribution of most *S. sclerotiorum* clones is therefore restricted geographically with little or no sharing of genotypes between different locations in the same country, resulting in genetically distinct subdivided populations as identified in Australia (Sexton and Howlett, 2004), UK (Clarkson et al., 2013) and USA (Malvárez et al., 2007). Although there is overwhelming support for homothallism and clonal reproduction in *S. sclerotiorum*, there has been some evidence for outcrossing and genetic exchange based on linkage disequilibrium measures (Atallah et al., 2004; Sexton and Howlett, 2004; Malvárez et al., 2007; Hemmati et al., 2009; Clarkson et al., 2013) and lack of association of molecular markers with mycelial compatibility group (MCG) (Atallah et al., 2004). More direct evidence of outcrossing has been through rare observations of sibling ascospores derived from a single apothecium belonging to more than one MCG (Atallah et al., 2004; Malvárez et al., 2007) and ascospore dimorphism (Ekins et al., 2006).

Although the population structure of *S. sclerotiorum* has been well-studied, there are fewer reports for related species such as *Sclerotinia minor* Jagger (Wu and Subbarao, 2006) *S. trifoliorum* Erikss. (Njambere et al., 2014) and none for *S. subarctica* nom. prov. *S. minor* has a reported host range of just over 90 species (Melzer et al., 1997) and like *S. sclerotiorum* is a major pathogen of lettuce (Wu and Subbarao, 2006). In one of the few population studies, Wu and Subbarao (2006) reported much lower levels of genetic diversity in *S. minor* compared with *S. sclerotiorum* based on MCGs for isolates from lettuce in California. In contrast to *S. sclerotiorum* and *S. minor, S. trifoliorum* is bipolar heterothallic (Uhm and Fujii, 1983) and has a more limited host range, being found mainly on cool-season forage and vegetable legumes (Willetts et al., 1980). A recent population study of *S. trifoliorum* on chickpea in California identified high levels of diversity based on MCGs and microsatellites (Njambere et al., 2014). Compared to the other *Sclerotinia* spp., *S. subarctica* was only identified relatively recently on the wild hosts yellow marsh marigold (*Caltha palustris*), dandelion (*Taraxacum* sp.), and northern wolfsbane (*Aconitum septentrionale*) in Norway (Holst-Jensen et al., 1998). It was first reported on horticultural crop hosts in Alaska, often in sympatry with *S. sclerotiorum* (Winton et al., 2006). A possible reason for this is that *S. subarctica* is difficult to distinguish from *S. sclerotiorum* as symptoms on plants are identical, and the two species look very similar in culture although *S. subarctica* generally forms larger sclerotia (Clarkson et al., 2010). Identification and designation as a new species was therefore based on three nucleotide substitutions in the ITS region and the absence of a 304 base group I intron in the large subunit (LSU) of the ribosomal RNA gene (Holst-Jensen et al., 1998). Since then, *S. subarctica* was first reported in the UK on meadow buttercup (*Ranunculus acris*) at a single location in England and pathogenicity demonstrated on oilseed rape (Clarkson et al., 2010). More recently, the pathogen has also been identified on a turnip rape crop (*Brassica rapa* subsp. *oleifera*) in Norway (Brodal et al., 2017). Little is known about the biology and epidemiology of *S. subarctica*, but one hypothesis is that it is more endemic to Northern latitudes (Winton et al., 2006). In addition, there have been no studies so far on *S. subarctica* population structure, although microsatellite markers have been published (Winton et al., 2007).

In a previous study, the population structure of *S. sclerotiorum* was examined in England and Wales (UK) for the first time using microsatellites and sequencing the intergenic spacer (IGS) region of the rRNA gene. (Clarkson et al., 2013). In total, 228 microsatellite haplotypes were identified within 384 isolates with one found at high frequency across different crop types and meadow buttercup. Of 14 IGS haplotypes identified, six were unique to buttercup and three were found at high frequency and were also present in *S. sclerotiorum* populations from Canada, the USA, and New Zealand published previously.

To date, *S. subarctica* has only been found on meadow buttercup at one location in England in sympatry with *S. sclerotiorum*, but it was hypothesized that *S. subarctica* may be more prevalent in the north of the UK and is likely to be found on crop plants (Clarkson et al., 2013). Hence one of the main aims of the present study was to sample and identify the species in further *Sclerotinia* populations from both crop plants and buttercup in England and Scotland. In addition, we sought to confirm that *S. subarctica* could still be isolated from the single location in Herefordshire (England) where it was first identified in samples collected in 2009 (Clarkson et al., 2010). For comparison, the relative incidence of *S. subarctica* and *S. sclerotiorum* was also examined for crop plants in Norway, a northern “neighbour” of the UK, where a high incidence of *S. subarctica* might be expected. Following identification of *S. subarctica*, a further aim was to genotype these isolates using microsatellites, hence providing a population structure analysis of this pathogen for the first time. Microsatellite and IGS data were also generated for isolates collected and identified as *S. sclerotiorum*, allowing population structure at a country scale to be examined for England, Scotland, and Norway. This added to the existing information generated in the previous study that focussed only on *S. sclerotiorum* populations from different locations in England. Finally, *S. sclerotiorum* isolates collected from Western Australia and genotyped using the same microsatellite markers and/or by IGS sequencing were also used as a geographically distant comparison with the UK and Norwegian populations.

## Materials and methods

### *Sclerotinia* isolates from different host plants

*Sclerotinia* species sclerotia were collected from a range of diseased crop plants comprising carrot (*Daucus carota*), cabbage (*Brassica oleracea*), celery (*Apium graveolens*), chinese cabbage (*Brassica rapa* subsp. *pekinensis*), camelina (*Camelina sativa*), Jerusalem artichoke (*Helianthus tuberosus*), lettuce (*Lactuca sativa*), oilseed rape (*Brassica napus* subsp. *napus*), potato (*Solanum tuberosum*), pumpkin (*Cucurbita pepo*), swede (*B. napus*), and turnip rape (*B. rapa* subsp. *oleifera*) from different locations in England, Scotland, and Norway between 2009 and 2013 (Table 1). For some crops, structured sampling was carried out whereby sclerotia were collected from infected plants at points at least 8 m apart along transects, with sclerotia collected from different points stored separately. For others, low levels of disease meant that this was not possible and sclerotia were collected from individual infected plants where found. Cultures of *Sclerotinia* were obtained from individual sclerotia by surface sterilizing them in a solution of 50% sodium hypochlorite (11–14% available chlorine, VWR International Ltd, UK) and 50% ethanol (v/v) for 4 min with agitation followed by two washes in sterile distilled water (SDW) for 1 min. The sclerotia were then bisected, placed on potato dextrose agar (PDA; Oxoid) and incubated at 20°C. After 2–3 days, agar plugs from the leading edge of actively growing mycelium were sub-cultured onto PDA and after ~6 weeks the mature sclerotia formed were stored both dry at 5°C and submerged in potato dextrose broth (PDB; Formedium, UK) amended with 10% glycerol (Sigma-Aldrich Company Ltd, UK) at −20°C. These stock sclerotia were used to initiate new cultures as required.

**Table 1:** **Origin and identity of *Sclerotinia* spp. isolates**.

**Location^1^**	**Year**	**Plant host**	**Total Isolates**	***S. sclerotiorum***		***S. subarctica***	
				**No. Isolates**	**No. genotyped^2^**	**No. Isolates**	**No. genotyped^3^**
**ENGLAND AND WALES**							
Blyth, Nottinghamshire (CA1)	2005	Carrot cv. Nairobi	32	32	32	0	0
Petworth, Sussex (LE1)	2005	Lettuce cv. Silverado	32	32	32	0	0
Preston Wynn, Herefordshire (OR1)	2005	Oilseed Rape cv. Winner	32	32	32	0	0
Holywell, Warwickshire (HO1)	2007	Meadow buttercup	32	32	32	0	0
Preston Wynn, Herefordshire (OR2)	2007	Oilseed Rape cv. Lioness	32	32	32	0	0
Deans Green, Warwickshire (DG1)	2008	Meadow buttercup	32	32	32	0	0
Holywell, Warwickshire (HO2)	2008	Meadow buttercup	32	32	32	0	0
Deans Green, Warwickshire (DG2)	2009	Meadow buttercup	32	32	32	0	0
Elan Valley, Powys (EV1)	2009	Meadow buttercup	32	32	32	0	0
Methwold, Norfolk (CE1)	2009	Celery cv. Victoria	32	32	32	0	0
Michaelchurch Escley1, Herefordshire (MI1)	2009	Meadow buttercup	50	44	32	16	15
Sutton St Nicholas, Herefordshire (PE1)	2009	Pea cv. Setchey	32	32	32	0	0
Vowchurch1, Herefordshire	2009	Oilseed Rape cv. unknown	40	40	32	0	0
Michaelchurch Escley1, Herefordshire	2010	Meadow buttercup	57	53	32	4	4
Michaelchurch Escley2, Herefordshire	2010	Meadow buttercup	32	32	32	0	0
Sutton Bridge, Lincolnshire	2010	Oilseed Rape cv. Catana	32	32	32	0	0
Upwood, Cambridgeshire	2010	Meadow buttercup	32	32	32	0	0
Vowchurch2, Herefordshire	2010	Oilseed Rape	32	32	32	0	0
Michaelchurch Escley, Herefordshire	2011	Meadow buttercup	40	25	24	15	15
Coxwold, North Yorkshire	2012	Carrot cv. Nairobi	32	32	0	0	0
Edwinstowe, Nottinghamshire	2012	Carrot cv. Nairobi	40	40	0	0	0
Total			749	714	600	35	34
**SCOTLAND**							
Coupar Angus, Perthshire	2010	Carrot cv. Nairobi	40	33	32	7	7
Bo'ness, West Lothian	2011	Meadow buttercup	44	43	32	1	1
Dunfermline, Fife	2011	Meadow buttercup	25	24	23	1	1
Bo'ness, West Lothian	2012	Meadow buttercup	45	42	0	3	3
Dunfermline, Fife	2012	Meadow buttercup	31	19	0	12	12
Isla Bend	2012	Potato	18	12	0	6	6
Meigle, Perthshire	2012	Pea	39	27	0	12	12
Muirhead, Lanarkshire	2012	Carrot	20	20	0	0	0
Tyninghame, East Lothian	2012	Swede	28	28	0	0	0
Eyemouth, Berwickshire	2013	Potato	34	16	0	18	18
Forfar, Angus	2013	Oilseed rape	15	15	0	0	0
Forfar, Angus	2013	Carrot	10	10	0	0	0
Glamis, Angus	2013	Carrot	12	12	0	0	0
Meigle, Perthshire	2013	Potato, Saxon	26	14	0	12	12
Redford, Angus	2013	Potato, Rooster	17	15	0	2	2
Total			404	330	87	74	74
**NORWAY**							
Buskerud	1993	Lettuce	1	1	1	0	0
Østfold	2012	Jerusalem Artichoke	1	0	0	1	1
Vestfold	2012	Swede	1	0	0	1	1
Akershus	2013	Camelina	13	13	13	0	0
Buskerud	2013	Lettuce, pumpkin	17	12	12	5	5
Hedmark	2013	Carrot	2	0	0	2	2
Nord-Trøndelag	2013	Lettuce, Chinese Cabbage, Potato	5	2	2	3	3
Oppland	2013	Turnip Rape	7	2	2	5	5
Østfold	2013	Jerusalem Artichoke, Celery Root	5	0	0	5	5
Rogaland	2013	Lettuce	39	18	18^*^	21	21
Vest-Agder	2013	Lettuce	6	1	1	5	5
Vestfold	2013	Lettuce	4	3	3	1	1
Vestfold	2013	Oilseed Rape	1	1	1	0	0
Total			102	53	53	49	49
**AUSTRALIA**							
Mount Barker	2004	Oilseed Rape	4	4	4	0	0
Walkaway	2004	Oilseed Rape	5	5	5	0	0
Binningup	2010	Carrot	1	1	1	0	0
East Chapman (3 sites)	2010	Oilseed Rape	10	10	10	0	0
Kendenup	2010	Oilseed Rape	3	3	3	0	0
Moonyoonooka (2 sites)	2010	Lupin	5	5	5	0	0
Mount Barker	2010	Oilseed Rape	5	5	5	0	0
Naragulu	2010	Oilseed Rape	4	4	4	0	0
Narra Tarra	2010	Oilseed Rape	5	5	5	0	0
Perth Metro area	2010	Cabbage	1	1	1	0	0
Walkaway (3 sites)	2010	Oilseed Rape	13	13	13	0	0
Walkaway	2010	Lupin	4	4	4	0	0
Eneabba	2013	Lupin	20	20	20^**^	0	0
Geraldton	2014	Oilseed Rape	20	20	20^**^	0	0
Mount Barker	2014	Oilseed Rape	16	16	16^**^	0	0
South Stirling, WA	2014	Oilseed Rape	15	15	15^**^	0	0
Total			131	131	131	0	0

1 *Locations followed by codes in brackets refer to data published previously by Clarkson et al. (2013)*.

2 Genotyped using microsatellites and sequencing of intergenic spacer (IGS) region of rRNA gene except

* one isolate genotyped by microsatellites only,

** *genotyped by IGS sequencing only*.

3 *Genotyped using microsatellites*.

Isolates of *Sclerotinia* spp. were also isolated from meadow buttercup in England and Scotland (Table 1) following the method described by Clarkson et al. (2013). Briefly, this was done by sampling flowers from five plants showing symptoms of infection, which were collected at up to 40 points at 10 m intervals along transects, with flowers from each plant stored separately. The flowers were then incubated on damp tissue paper in sealed plastic boxes at room temperature (~22°C) for 4 weeks. Sclerotia formed on the damp tissue paper were then picked off and cultured as described previously.

Isolates of *S. sclerotiorum* collected between 2009 and 2014 were also obtained from sclerotia collected from infected lupin and oilseed rape from the northern and southern agricultural regions of Western Australia in addition to “standard” isolates from oilseed rape (collected 2004), cabbage, and carrot (collected 2010) (Ge et al., 2012). These were cultured and stored as described previously.

### DNA extraction and molecular identification of *S. sclerotiorum* and *S. subarctica*

*Sclerotinia* spp. cultures were initiated from stock sclerotia and incubated on PDA at 20°C for 3–4 days to produce actively growing colonies. Three agar plugs were taken from the leading edge, placed into Petri dishes containing half strength PDB, and incubated at 20°C for 3 days. The agar plugs were then removed and the mycelial mat washed twice in sterilized reverse osmosis (RO) water and blotted dry on tissue (KimTech; Kimberly-Clark Ltd, UK) before being freeze-dried overnight. Genomic DNA was extracted from the freeze-dried mycelium using a DNeasy Plant Mini Kit (Qiagen Ltd, UK) following the manufacturer's protocol.

*S. subarctica* and *S. sclerotiorum* isolates were distinguished by PCR amplification of the large subunit of the ribosomal DNA (LSU), where a large (304 bp) intron is absent in *S. subarctica* compared to *S. sclerotiorum* (Holst-Jensen et al., 1998). The 25 μl PCR reaction mixture consisted of 1 x REDTaq ReadyMix PCR reaction mix (Sigma-Aldrich, UK), LR5 and LROR primers (0.4 μmol L^−1^; (Vilgalys and Hester, 1990) and ~10 ng DNA template. Thermal cycling parameters were 94°C for 2 min; 35 cycles of 94°C for 60 s, 52°C for 60 s, 72°C for 60 s; 72°C for 10 min and then a hold at 12°C. PCR products were visualized on a 1.5% agarose gel with a DNA ladder (EasyLadder I, Bioline Reagents Ltd, UK). Isolates associated with the smaller sized amplicons were identified as *S. subarctica* and this was further confirmed by PCR amplification and sequencing of the rRNA ITS region. Here, the PCR reaction mixture of 25 μl consisted of 1 x REDTaq ReadyMix PCR reaction mix (Sigma-Aldrich, UK), modified standard ITS primers (White et al., 1990) for *S. sclerotiorum* ITS2AF (TCGTAACAAGGTTTCCGTAGG) and ITS2AR (CGCCGTTACTGAGGTAATCC; 0.4 μmol L^−1^) and approximately 10 ng DNA template. Thermal cycling parameters were 94°C for 2 min; 40 cycles of 94°C for 15 s, 59°C for 15 s, 72°C for 30 s; 72°C for 10 min and then a hold at 12°C. PCR products were visualized on a 1.5% agarose gel to confirm amplification, purified using the QIAquick PCR purification kit (Qiagen, UK), and sequenced (ITS2AF/ITS2AR primers) by GATC Biotech (Germany). ITS sequences obtained for all the *S. subarctica* isolates were aligned using the ClustalW algorithm implemented in MEGA v6 (Tamura et al., 2013) and sequence identity was confirmed by BLASTn analysis.

### Molecular characterization of *Sclerotinia subarctica* isolates using microsatellites

Isolates identified as *S. subarctica* were characterized using eight microsatellite markers in two multiplexed PCR reactions (loci MS01, MS03, MS06, MS08 and MS02, MS04, MS05, MS07) with fluorescent-labeled primer pairs (Applied Biosystems, UK) as developed by Winton et al. (2007). Each PCR reaction mixture of 20 μl consisted of 1 x QIAGEN Multiplex PCR Master Mix, 0.5 x Q solution, primer mix (0.4 μmol L^−1^) and ~10 ng DNA template. Thermal cycling parameters were 95°C for 15 min; 35 cycles of 94°C for 30 s, 55°C for 90 s, 69°C for 75 s; 60°C for 30 min and then a hold at 12°C. PCR products were visualized on a 1.5% agarose gel to confirm amplification and two separate PCR amplifications per locus were carried out for each isolate to ensure reproducibility of results. The size of all PCR amplicons was determined by Eurofins (Germany) using an ABI 3130xl genetic analyser and allele sizes assigned using GENEMARKER (Version 1.6; SoftGenetics, USA). FLEXIBIN (Amos et al., 2007) was then used to bin allele sizes and estimate the relative number of repeats at each locus.

### Molecular characterization of *Sclerotinia Sclerotiorum* isolates using microsatellites and IGS sequencing

Isolates identified as *S. sclerotiorum* were characterized using eight microsatellite markers (Sirjusingh and Kohn, 2001) in two multiplexed PCR reactions (loci 13-2, 17-3, 55-4, 110-4, 114-4 and 7-2, 8-3, 92-4) using fluorescent-labeled primer pairs (Applied Biosystems, UK). The PCR reaction mixture of 10 μl consisted of 1 x QIAGEN Multiplex PCR Master Mix, 0.5 x Q solution, forward and reverse primer pairs (0.2 μmol L^−1^) and ~10 ng DNA template. Thermal cycling parameters were 95°C for 15 min; 35 cycles of 94°C for 30 s, 55°C for 90 s, 69°C for 75 s; 69°C for 75 s and then a hold at 12°C. PCR products were visualized on a 1.5% agarose gel to confirm amplification and two separate PCR amplifications per locus were carried out for each isolate to ensure reproducibility of results. The size of all PCR amplicons was determined by Eurofins (Germany) using an ABI 3130xl genetic analyser and allele sizes assigned using GENEMARKER (Version 1.6; SoftGenetics, USA). FLEXIBIN (Amos et al., 2007) was then used to bin allele sizes and estimate the number of repeats for each locus.

*S. sclerotiorum* isolates were also characterized by sequencing part of the IGS region of the rRNA gene where PCR primers IGS2F (TTACAAAGATCCTCTTTCCATTCT) and IGS2R (GCCTTTACAGGCTGACTCTTC) (Clarkson et al., 2013) were used to amplify an 834 bp (approx.) fragment. The PCR reaction mixture of 25 μl consisted of 0.5 x REDTaq ReadyMix PCR reaction mix (Sigma-Aldrich, UK), IGS2F and IGS2R primers (4 μmol L^−1^) and ~10 ng DNA template. Thermal cycling parameters were 94°C for 2 min, 40 cycles of 94°C for 30 s, 57°C for 30 s, 72°C for 2 min followed by 72°C for 10 min and a hold of 12°C. PCR products were visualized on a 1.5% agarose gel to confirm amplification, purified using the QIAquick PCR purification kit (Qiagen, UK), and sequenced (IGS2F/IGS2R primers) by GATC Biotech (Germany). IGS primers did not consistently amplify an initial selection of *S. subarctica* isolates and hence sequences were not generated for this species.

### Analysis of *Sclerotinia sclerotiorum* and *Sclerotinia subarctica* microsatellite data

ARLEQUIN (Excoffier et al., 2005) was used to determine the haplotype frequency of *S. subarctica* /*S. sclerotiorum* isolates for each country based on the relative number of repeats at each microsatellite locus and to identify shared haplotypes. Genodive (Meirmans and van Tienderen, 2004) was used to calculate gene diversity (*Hs*) for each locus (Nei, 1987) and the average across all loci and furthermore generate clonal (haplotype) diversity statistics for each Sclerotinia species in the different countries. These comprised haplotype diversity (*div*) (Nei, 1987) and a corrected form of the Shannon-Wiener Index (*shc*). The former is based on frequencies of haplotypes in each population while the latter is based on the abundance and evenness of haplotypes. While *div* is independent of sample size (Nei, 1987) the Shannon index is prone to bias when comparing unequal sample sizes. However, the corrected form calculated in Genodive accounts for this through a non-parametric approach which uses unequal probability sampling theory (Chao and Shen, 2003). Calculations of both *div* and *shc* as implemented in Genodive also included a jackknife approach to estimate the relationship between sample size and diversity and in all cases, the variance in diversity decreased with increasing population size and leveled off below the population size sampled. A bootstrap test (1,000 permutations) also implemented in Genodive allowed us to test if *Sclerotinia* populations from different countries differed in their haplotype diversity as measured by *div* and *shc*. POPPR (Kamvar et al., 2014) was used to calculate multilocus indices of disequilibrium; the index of association *I*_*A*_ and the index r_d_ which accounts for the number of loci (Agapow and Burt, 2001). ARLEQUIN was used to test for subdivision for both *S. sclerotiorum* and *S. subarctica* populations from different countries and was estimated through pairwise comparisons of *R*_ST_ (Slatkin, 1995), a statistic which uses a stepwise mutation model which has been widely implemented for microsatellite data [including for *S. sclerotiorum* e.g., Aldrich-Wolfe et al. (2015)], with significance tested by permuting (1,023) haplotypes between populations. The analysis for *S. sclerotiorum* included data sets for the 12 English *S. sclerotiorum* populations (384 isolates, Table 1) published previously (Clarkson et al., 2013).

*S. subarctica* and *S. sclerotiorum* microsatellite data were subjected to Bayesian population structure analyses using STRUCTURE v2.3.3 (Pritchard et al., 2000), an approach used previously for *Sclerotinia* spp. (Attanayake et al., 2013, 2014; Njambere et al., 2014). The markers used in this study map to different chromosomes of the *S. sclerotiorum* reference genome (Amselem et al., 2011) with the exception of 7-2 and 114-4 which both map to chromosome 4 and 13-2 and 110-4 which both map to chromosome 6. These pairs that map to the same chromosomes were shown by Attanayake et al. (2014) to be of sufficient distance for LD to decay (Bastien et al., 2014) and therefore satisfy the assumptions of STRUCTURE. A burn-in period of 300,000 Markov Chain Monte Carlo iterations and a 300,000 run-length was implemented using an admixture model and correlated allele frequencies for *K* values between 1 and 6. For each simulated cluster for *K* = 1–6, four runs were repeated independently for consistency. For the *S. sclerotiorum* data, this was followed by a second analysis using a burn-in period of 500,000 Markov Chain Monte Carlo iterations and a 500,000 run-length implemented using an admixture model and correlated allele frequencies for *K* values between 3 and 5. Again, for each simulated cluster for *K* = 3–5, four runs were repeated independently. For both *Sclerotinia* species, the python script structure Harvester.py v0.6.92 (Earl and vonHoldt, 2012) was then used to summarize the STRUCTURE output, producing Δ*K* values using the Evanno method (Evanno et al., 2005) to estimate the most likely underlying *K*. Replicate simulations of cluster membership (q-matrices) at *K* = 4 for *S. sclerotiorum* isolates and *K* = 2 for *S. subarctica* isolates were used as input for CLUMPP_OSX.1.1.2 (Jakobsson and Rosenberg, 2007) using the Fullsearch algorithm, with weighted H and the G similarity statistic. Summarized cluster membership matrices (*q*-values) for both individuals and populations were then visualized using distruct_OSX1.1 (Rosenberg, 2004).

The microsatellite data represented a multivariate dataset and to reduce the complexity of the data, principal component analyses (PCA) were used to complement the STRUCTURE analyses. For both *Sclerotinia* species, analyses were performed first on the microsatellite repeat size data, and then on an allele score matrix constructed by subdividing each microsatellite into repeat size categories, then scoring if the repeat size is present or absent in each isolate, forming a binary score data matrix. Principal components were estimated using the singular value decomposition method implemented in R v3.2.3 (R-Development-Core-Team, 2015) using the built in “prcomp” function and the package FactoMineR v1.31.4 (Lê et al., 2008). Scatter plots of the component scores were produced using ggplot2 (Wickham, 2016), with ellipses representing Euclidean distance from the center (confidence level = 0.95) of each cluster.

### Analysis of IGS sequence data for *Sclerotinia sclerotiorum*

*S. sclerotiorum* IGS sequences were aligned using the ClustalW algorithm implemented in MEGA v6 (Tamura et al., 2013) and DNASP v. 5 (Librado and Rozas, 2009) was used to identify haplotypes based on sequence differences (omitting indels), calculate haplotype diversity and also used to examine subdivision between populations from different countries using pairwise comparisons of the nearest neighbor statistic (Snn) (Hudson, 2000) with significance calculated with 1,000 permutations. Again, data sets for the 12 English *S. sclerotiorum* populations (384 isolates) published previously (Clarkson et al., 2013) were also included in the analysis. A median joining network of IGS haplotypes (Bandelt et al., 1999) which included sequence data from Canada, New Zealand, Norway, and the USA (Carbone and Kohn, 2001a) was constructed using NETWORK v. 4.6 (Fluxus Technology, USA) for all the datasets from England, Scotland, Norway, and Australia.

## Results

### Molecular identification and frequency of *Sclerotinia subarctica*

A total of 843 *Sclerotinia* isolates were collected from crop plants and buttercup in England (337), Scotland (404), and Norway (102). Identification through amplification of the LSU rDNA showed that 142 of these were *S. subarctica* (Table 1) with the proportion rising to 157 of 1,255 isolates when previous data from England (412 isolates) was included (Table 1). *S. subarctica* was detected on a wide range of host plants and there was increased incidence in samples collected from Scotland and Norway. In England, only 35 of 749 *Sclerotinia* isolates (4.7%) were *S. subarctica* and these were all isolated from a single buttercup meadow in Herefordshire over 3 years (2009–2011). In Scotland however, 74 of 404 isolates (18.3%) were *S. subarctica* and were found in the majority of crops, locations and years between 2010 and 2013. In Norway, 49 of 102 *Sclerotinia* isolates (48.0%) collected in 2012/2013, again from a wide range of crop types and locations were identified as *S. subarctica*. Identity of all *S. subarctica* isolates was further confirmed by sequencing of the rRNA ITS region and all sequences were identical to that previously deposited in Genbank (GU018183) (Clarkson et al., 2010).

### Molecular characterization of *Sclerotinia subarctica* isolates using microsatellites

**Figure 1:**
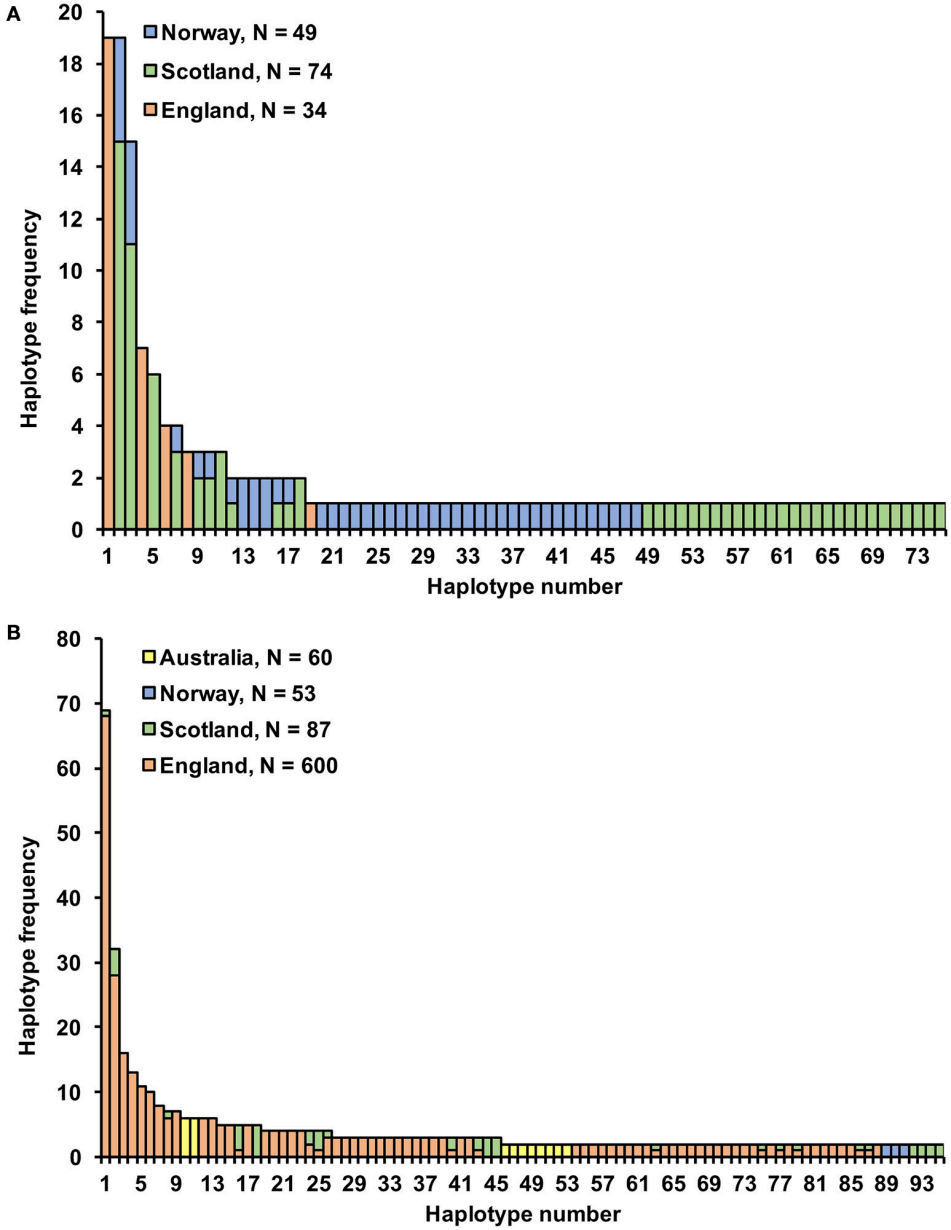
**Microsatellite haplotype frequency for (A)** 157 *S. subarctica* isolates from England, Scotland, and Norway; **(B)** 800 *S. sclerotiorum* isolates from England, Scotland, Norway, and Australia for 95 haplotypes comprising more than one isolate.

Microsatellite analysis of the *S. subarctica* isolates resulted in 5 to 10 polymorphic alleles per locus, with loci MS01, MS02, MS03, MS04, MS05, MS06, MS07, and MS08 having 10, 6, 5, 10, 5, 7, 7, and 6 alleles respectively (Table 2). Two of the loci (MS02 and MS04) were monomorphic for the isolates from England. Overall, 75 microsatellite haplotypes were identified within the 157 *S. subarctica* isolates genotyped from England, Scotland, and Norway (Figure 1A). The number of different haplotypes as a proportion of the total number of isolates differed between countries, England having fewer haplotypes (14%; 5 haplotypes within 34 isolates) compared to Scotland (51%; 38 haplotypes within 74 isolates) and Norway (82%, 40 haplotypes within 49 isolates). Over all the 75 *S. subarctica* haplotypes, 18 were represented by more than one isolate with eight shared between Scotland and Norway but none shared between England and Scotland or England and Norway (Figure 1A). A few haplotypes were sampled more frequently than the rest. The most prevalent haplotypes in England were haplotypes 1 and 4 (19 and 7 isolates respectively; Table 3) and both were found in each of the 3 years sampling at the buttercup meadow in Herefordshire. Haplotypes 2 and 3 were most prevalent for both Scotland and Norway (19 and 15 isolates respectively; Table 3) and were represented in samples from potato, buttercup and swede (Scotland) and carrot, lettuce and swede (Norway). Haplotype diversity measures *div* and *shc* were significantly lower for the *S. subarctica* isolates from England compared to Scotland and Norway (Table 4; *P* < 0.001) as was the diversity in Scotland compared to Norway (*P* < 0.05). The index of association *I*_*A*_ for *S. subarctica* microsatellite data ranged between 0.57 (Norway) and 2.9 (England) while r_d_ was between 0.08 (Norway) and 0.58 (England). Significance testing showed that the hypothesis of random mating was rejected in all cases (*P* < 0.001; Table 4). This was also true when clone-corrected data was used in the analysis (Table 4). There was also evidence of subdivision between the *S. subarctica* populations from the three different countries with the *R*_*ST*_ fixation index statistic highly significant (*P* < 0.0001) for pairwise combinations of England/Scotland (*R*_ST_ = 0.637) and England/Norway (*R*_ST_ = 0.591). However, this was less significant (*P* < 0.05) for the Scotland/Norway combination (*R*_ST_ = 0.030) as might be expected given some sharing of haplotypes.

**Table 2:** **Summary of microsatellite data for *S. subarctica* isolates from England, Scotland, and Norway**.

**Locus^1^**	**Allele size range**			**Total alleles**	**Number of alleles**			**Number of private alleles**			**Gene diversity (*Hs*)^2^**		
	**ENG**	**SCO**	**NOR**		**ENG**	**SCO**	**NOR**	**ENG**	**SCO**	**NOR**	**ENG**	**SCO**	**NOR**
MS01	129–146	128–184	128–161	10	3	6	7	1	3	2	0.358	0.515	0.677
MS02	174	161–193	162–180	6	1	6	4	0	0	2	0.000	0.352	0.290
MS03	193–203	170–194	184–193	5	2	3	3	1	1	1	0.166	0.442	0.371
MS04	189	175–200	178–211	10	1	8	5	0	2	5	0.000	0.733	0.742
MS05	320–346	317–333	317–331	5	3	4	2	1	0	0	0.358	0.592	0.505
MS06	378–424	348–416	370–408	7	4	6	4	1	0	2	0.629	0.417	0.616
MS07	372–389	362–374	362–382	7	3	2	5	2	3	0	0.597	0.151	0.330
MS08	378–394	371–383	371–391	6	3	2	5	1	2	0	0.597	0.151	0.297
Mean				7.0	2.5	4.6	4.4	0.9	1.4	1.5	0.338	0.419	0.479

1 *Loci as defined by Winton et al. (2007)*.

2 *Nei's gene diversity (Nei, 1987)*.

**Table 3:** **Location, year, host, and frequency of most common *S. subarctica* microsatellite haplotypes in England, Scotland, and Norway**.

**Location**	**Year**	**Host/crop**	**Microsatellite haplotype^1^**			
			**hap 1**	**hap 2**	**hap 3**	**hap 4**
**ENGLAND**						
Michaelchurch Escley, Herefordshire	2009	Meadow buttercup	9	0	0	3
Michaelchurch Escley, Herefordshire	2010	Meadow buttercup	2	0	0	2
Michaelchurch Escley, Herefordshire	2011	Meadow buttercup	8	0	0	2
Total			19	0	0	7
**SCOTLAND**						
Eyemouth, Berwickshire	2013	Potato	0	3	8	0
Isla Bend	2012	Potato	0	6	0	0
Dunfermline, Fife	2012	Meadow buttercup	0	3	0	0
Meigle, Perthshire	2012	Pea	0	3	3	0
Total			0	15	11	0
**NORWAY**						
Rogaland	2013	Lettuce	0	1	1	0
Vest-Agder	2013	Lettuce	0	1	2	0
Vestfold	2013	Swede	0	1	0	0
Hedmark	2013	Carrot	0	1	1	0
Total			0	4	4	0
Grand total			19	19	15	7

1 *Two microsatellite haplotypes most prevalent in England (1 and 4), Scotland, and Norway (2 and 3)*.

**Table 4:** **Diversity statistics and disequilibrium measures for *S. subarctica* isolates from England (ENG), Scotland (SCO), and Norway (NOR) based on microsatellite data**.

	**No. isolates**	**No. haplotypes**	**No. unique haplotypes^1^**	***shc*^2^**	***div*^3^**	***I_*A*_*^4^ all clones**	***I_*A*_*^4^ clone corrected**	***r*^4^_*d*_ all clones**	***r*^4^_*d*_ clone corrected**
ENG	34	5	5	0.560	0.642	2.874^***^	–	0.581^***^	–
SCO	74	38	30	1.610	0.932	0.779^***^	0.259^**^	0.113^***^	0.040^**^
NOR	49	40	32	2.096	0.987	0.567^***^	0.485^***^	0.081^***^	0.070^***^

1 *Haplotypes not found in any other country*.

2 *Shannon-Wiener Diversity corrected for sample size (Chao and Shen, 2003)*.

3 *Haplotype diversity corrected for sample size (Nei, 1987)*.

4 Index of Association (I_A_) and related measure r_d_ (Agapow and Burt, 2001) for all clones and clone corrected data.

*** (P < 0.001);

** *(P < 0.006). Not calculated for clone corrected data from England due to small number of haplotypes*.

**Figure 2:**
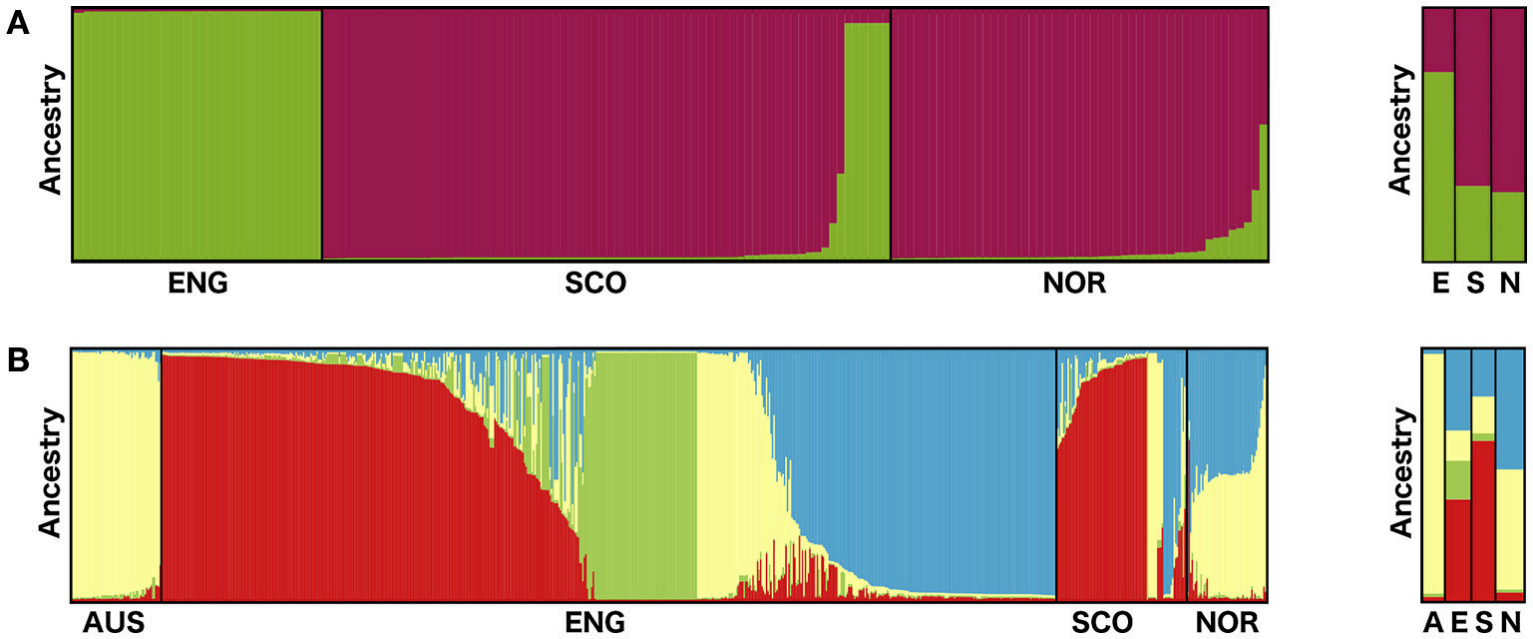
**Bar plots showing the assignment of (A)** *S. subarctica* isolates (*N* = 157) from England (ENG, *N* = 34), Scotland (SCO, *N* = 74), and Norway (NOR, *N* = 49) to two ancestral populations and **(B)** *S. sclerotiorum* isolates (*N* = 800) from Australia (AUS, *N* = 60), England (*N* = 600), Scotland (*N* = 87), and Norway (*N* = 53) to three ancestral populations from STRUCTURE analysis of microsatellite data. Assignment to populations is based on q values for each isolate (*S. subarctica* q1 green, q2 purple; *S. sclerotiorum* q1 red, q2 green, q3 yellow, q4 blue) and population q values for each country (A, Australia; E, England; S, Scotland; N, Norway.).

**Figure 3:**
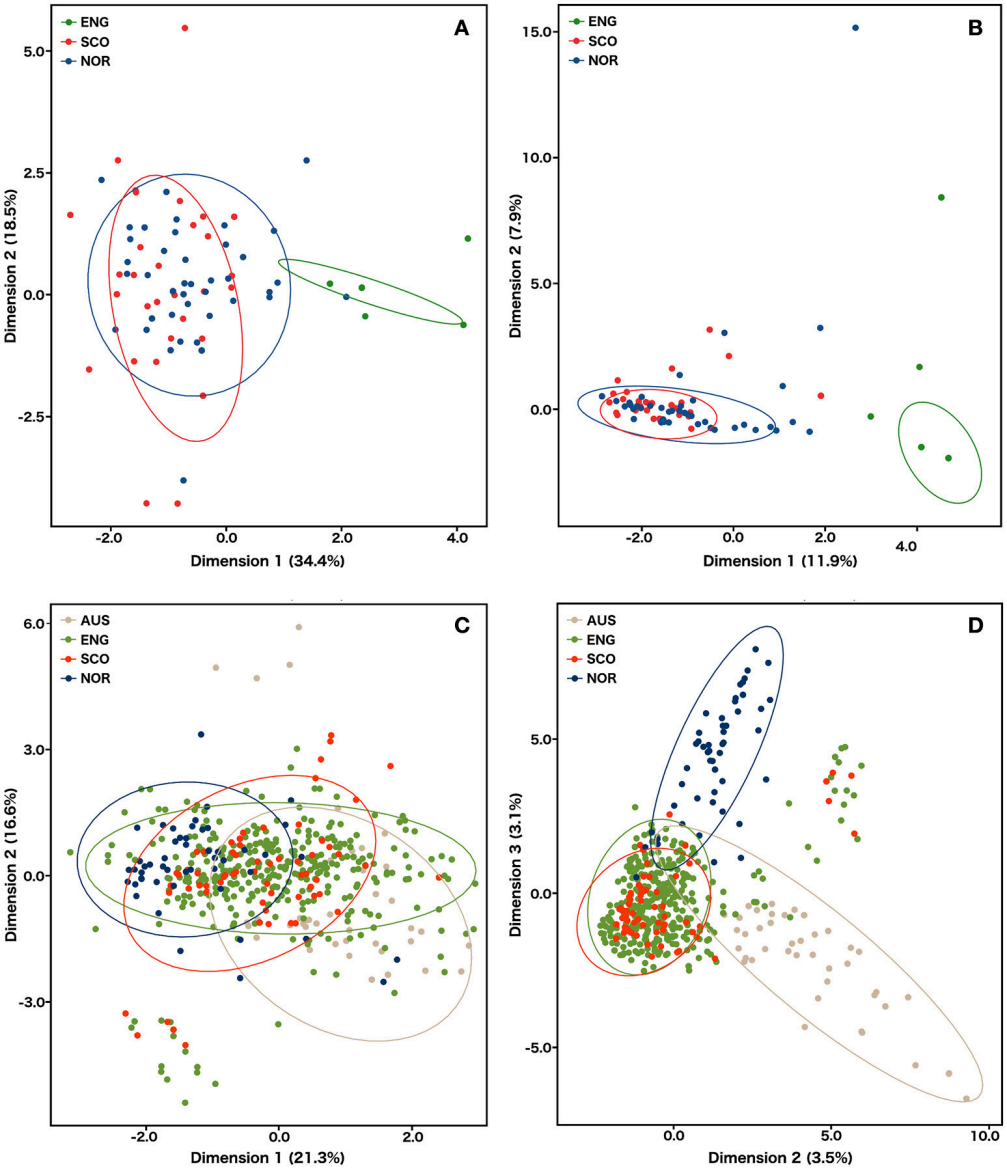
**Principal component analysis of microsatellite data for *S. subarctica* isolates from England (N = 34), Scotland (*N* = 74), and Norway (*N* = 49) and *S. sclerotiorum* isolates from Australia (*N* = 60), England (*N* = 600), Scotland (*N* = 87), Norway (*N* = 53)**. **(A)** *S. subarctica* individuals factor map estimated using microsatellite repeat number; **(B)** *S. subarctica* individuals factor map estimated using an allele presence binary matrix; **(C)** *S. sclerotiorum* individuals factor map estimated using microsatellite repeat number; **(D)** *S. sclerotiorum* individuals factor map estimated using an allele presence binary matrix. Individual isolates are colored by geographic origin. Ellipses represent the Euclidean distance from the center (confidence level = 0.95) of each cluster.

The Bayesian cluster analysis of the *S. subarctica* microsatellite data using STRUCTURE suggested two genetically distinct ancestral populations (*K* = 2, being the value associated with the highest value of Δ*K*). Examining the probability of each isolate belonging to either of the two sub-populations described by the membership coefficient matrix, all 34 of the isolates from buttercup collected from the single site in Herefordshire (Michaelchurch Escley) in England between 2009 and 2012 were assigned to cluster *q*1, whereas all the isolates from Norway (49) and 68 of the 74 (92%) isolates from Scotland were assigned to *q*2 (Figure 2A). This suggests that the majority of the isolates from Norway and Scotland share a common ancestry. However, six of the Scottish isolates (SC52-SC57), which were all collected from meadow buttercup at a site near Dunfermline (Fife) in 2012 were assigned to *q*1 and hence appear to share ancestry with the English isolates. The reduced genetic diversity in the English isolates and the shared ancestry of Scottish and Norwegian *S. subarctica* isolates was also evident in the principal component analysis using either microsatellite repeat number or the binary matrix. The first principal component clearly distinguished Scottish and Norwegian isolates from the English isolates and the reduced genetic variation of the English isolates was indicated by the reduced space they occupied in the PCA map, particularly in dimension 2 (Figures 3A,B).

### Molecular characterization of *Sclerotinia sclerotiorum* isolates using microsatellites

Microsatellite analysis of the *S. sclerotiorum* isolates resulted in 7 to 19 polymorphic alleles per locus, with loci 7-2, 8-3, 13-2, 17-3, 55-4, 92-4, 110-4, 114-4 having 11, 15, 19, 20, 20, 7, 8, and 2 alleles respectively (Table 5). Overall, 484 microsatellite haplotypes were identified within the 800 *S. sclerotiorum* isolates that were genotyped (Figure 1B). In England, 343 haplotypes were found within 600 isolates (57%), with 64 (74%), 50 (94%), and 42 haplotypes (70%) identified within 87, 53, and 60 isolates from Scotland, Norway, and Australia respectively. Over all 800 *S. sclerotiorum* haplotypes, 95 were represented by more than one isolate while 15 were shared between England and Scotland (Figure 1B). No haplotypes were shared between any other countries. In each country, there were a small number of haplotypes sampled more frequently than the rest. In England, 68 isolates representing the most prevalent haplotype 1 were found in the majority of hosts and locations sampled and a further single representative was identified in Scottish buttercup (Figure 1B, Table 6). The second most prevalent haplotype 2 in England comprised 25 isolates from different buttercup meadows but was only identified in a single crop location (lettuce, three isolates). Haplotype 2 was also found in two buttercup meadows (four isolates, 2011; Table 6) in Scotland. However, the most prevalent haplotype 18 in Scotland was represented by five isolates found in two different buttercup locations (2011) and in carrot (2010) (Table 6), but was not present in England. Haplotype 16 comprising four isolates from buttercup and carrot in Scotland (Table 6) was also found in one buttercup meadow in England (Holywell, 2008). In Norway, the most prevalent haplotypes were haplotype 89, 90, and 91 each of which was represented by just two isolates from oilseed rape, pumpkin, and turnip rape hosts while the two most prevalent haplotypes 10 and 11 in Australia both comprised six isolates each from oilseed rape and lupin from different locations (Table 6).

**Table 5:** **Summary of microsatellite data for *S. sclerotiorum* isolates from England (ENG), Scotland (SCO), Norway (NOR), and Australia (AUS)**.

**Locus^1^**	**Allele size range**				**Total alleles**	**Number of alleles**				**Number of private alleles**				**Gene diversity (*Hs*)^2^**			
	**ENG**	**SCO**	**NOR**	**AUS**		**ENG**	**SCO**	**NOR**	**AUS**	**ENG**	**SCO**	**NOR**	**AUS**	**ENG**	**SCO**	**NOR**	**AUS**
7–2	159–175	170–173	156–172	162–236	11	5	2	4	6	3	0	2	4	0.593	0.332	0.491	0.583
8–3	228–260	252–256	246–252	252–270	15	9	3	4	8	4	0	2	4	0.617	0.326	0.557	0.637
13–2	278–382	300–359	289–349	278–373	19	18	8	7	10	3	0	0	1	0.791	0.701	0.758	0.868
17–3	342–394	341–401	339–368	336–377	20	16	11	8	9	3	0	2	2	0.745	0.761	0.751	0.868
55–4	149–217	154–238	155–220	157–185	20	15	10	9	4	6	2	1	0	0.702	0.717	0.821	0.436
92–4	370–381	370–379	369–379	373–379	7	7	5	5	4	1	0	0	0	0.564	0.509	0.525	0.666
110–4	352–387	352–387	368–383	368–383	8	8	7	4	3	1	0	0	0	0.717	0.495	0.568	0.579
114–4	345–421	349–388	350–390	356–408	21	20	10	7	9	6	0	0	1	0.835	0.810	0.750	0.847
Mean					15.1	12.3	7.0	6.0	6.6	3.4	0.3	0.9	1.5	0.695	0.581	0.653	0.686

1 *Loci as defined by Sirjusingh and Kohn (2001)*.

2 *Nei's gene diversity (Nei, 1987)*.

**Table 6:** **Location, year, host, and frequency of the most prevalent *S. sclerotiorum* microsatellite haplotypes in England (Hap 1, 2, 3), Scotland (Hap 2, 16, 18), Norway (Hap 89, 90, 91), and Australia (Hap 10, 11)**.

	**Year**		**Hap 1**	**Hap 2**	**Hap 3**	**Hap 16**	**Hap 18**
**ENGLAND AND WALES**							
Blyth, Nottinghamshire	2005	Carrot	5	0	0	0	0
Petworth, Sussex	2005	Lettuce	4	3	2	0	0
Preston Wynn, Herefordshire	2005	Oilseed Rape	3	0	0	0	0
Holywell, Warwickshire	2007	Meadow buttercup	6	1	0	0	0
Preston Wynn, Herefordshire	2007	Oilseed rape	3	0	0	0	0
Deans Green, Warwickshire	2008	Meadow buttercup	2	10	1	0	0
Holywell, Warwickshire	2008	Meadow buttercup	4	0	0	1	0
Deans Green, Warwickshire	2009	Meadow buttercup	5	3	0	0	0
Elan Valley, Powys	2009	Meadow buttercup	0	2	0	0	0
Methwold, Norfolk	2009	Celery cv. Victoria	1	0	8	0	0
Michaelchurch Escley, Herefordshire	2009	Meadow buttercup	4	2	0	0	0
Sutton St Nicholas, Herefordshire	2009	Pea cv. Setchey	5	0	0	0	0
Vowchurch, Herefordshire	2009	Oilseed Rape	2	0	3	0	0
Michaelchurch Escley 1, Herefordshire	2010	Meadow buttercup	0	4	0	0	0
Michaelchurch Escley 2, Herefordshire	2010	Meadow buttercup	3	1	0	0	0
Sutton Bridge, Lincolnshire	2010	Oilseed Rape	9	0	0	0	0
Upwood, Cambridgeshire	2010	Meadow buttercup	8	0	0	0	0
Vowchurch, Herefordshire	2010	Oilseed Rape	3	0	1	0	0
Michaelchurch Escley, Herefordshire	2011	Meadow buttercup	1	2	1	0	0
Total			68	28	16	1	0
**SCOTLAND**							
Coupar Angus, Perthshire	2010	Carrot cv. Nairobi	0	0	0	1	1
Bo'ness, West Lothian	2011	Meadow buttercup	1	3	0	2	2
Dunfermline, Fife	2011	Meadow buttercup	0	1	0	1	2
Total			1	4	0	4	5
			**Hap 89**	**Hap 90**	**Hap 91**		
**NORWAY**							
Buskerud (2 sites; A, B)	2013	Lettuce (A), pumpkin (B)	1 (B)	0	0		
Oppland	2013	Turnip rape	0	2	2		
Vestfold	2013	Oilseed Rape	1	0	0		
Total			2	2	2		
			**Hap 10**	**Hap 11**			
**AUSTRALIA**							
Walkaway	2004	Oilseed Rape	0	1			
East Chapman (3 sites; A, B,C)	2010	Oilseed Rape	2 (B)	1 (B)			
Moonyoonooka (2 sites; A, B)	2010	Lupin	2 (B)	1 (A)			
Walkaway (3 sites; A, B, C)	2010	Oilseed Rape	2 (A)	3 (A, B)			
Total			6	6			

Haplotype diversity measures of *div* and *shc* for the *S. sclerotiorum* microsatellite data were generally high for England Scotland and Norway but lower for the Australian isolates (Table 7) but this was only significant for the Norway/Australia pairwise combination for *div* (*P* < 0.05). However, pairwise comparisons showed that *shc* was significantly greater for England compared to Scotland (*P* < 0.05) and Australia (*P* < 0.01) while *shc* was significantly greater in Norway compared to all the other three countries (*P* < 0.001). There was also evidence of genetic differentiation between the *S. sclerotiorum* populations from the four different countries with the *R*_*ST*_ fixation index statistic highly significant (*P* < 0.0001) for pairwise combinations of England/Norway (*R*_ST_ = 0.094), England/Australia (*R*_ST_ = 0.186), Scotland/Norway (*R*_ST_ = 0.068), Scotland/Australia (*R*_ST_ = 0.152), and Norway/Australia (*R*_ST_ = 0.198). Differentiation between England/Scotland was less significant (*P* < 0.01, *R*_ST_ = 0.015). The index of association *I*_*A*_ for *S. sclerotiorum* microsatellite data ranged between 0.26 (Norway) and 0.95 (Australia) while r_d_ was between 0.04 (Norway) and 0.14 (Australia). Significance testing showed that the hypothesis of random mating was rejected in all cases (*P* < 0.001; Table 7). This was also the case when clone-corrected data was used in the analysis (Table 7).

**Table 7:** **Diversity statistics and disequilibrium measures for *S. sclerotiorum* isolates from England (ENG), Scotland (SCO), Norway (NOR), and Australia (AUS) based on microsatellite data**.

	**No. isolates**	**No. haplotypes**	**No. unique haplotypes^1^**	***shc*^2^**	***div*^3^**	***I_*A*_*^4^ all clones**	***I_*A*_*^4^ clone corrected**	***r*^4^_*d*_ all clones**	***r*^4^_*d*_ clone corrected**
ENG	600	343	328	2.536	0.982	0.762^***^	0.241^***^	0.110^***^	0.035^***^
SCO	87	64	49	2.133	0.990	0.662^***^	0.542^***^	0.095^***^	0.078^***^
NOR	53	50	50	2.652	0.998	0.257^***^	0.181^**^	0.037^***^	0.026^**^
AUS	60	42	42	1.904	0.979	0.945^***^	0.579^***^	0.138^***^	0.090^***^

1 *Haplotypes not found in any other country*.

2 *Shannon-Wiener Diversity corrected for sample size (Chao and Shen, 2003)*.

3 *Haplotype diversity corrected for sample size (Nei, 1987)*.

4 Index of Association (I_A_) and related measure r_d_ (Agapow and Burt, 2001) for all clones and clone corrected data.

*** (P < 0.001);

** *(P < 0.006)*.

The Bayesian cluster analysis of the *S. sclerotiorum* microsatellite data using STRUCTURE suggested the number of genetically distinct ancestral populations was best represented by *K* = 4 clusters, the value associated with the highest value of Δ*K* (Figure 2B) The majority of English isolates were assigned to cluster *q*1 (251, 42%) and *q*4 (189, 32%) followed by *q*2 (84, 14%) and to a lesser extent *q*3 (53 isolates, 9%). Similarly, most of the Scottish isolates were also assigned to cluster *q*1 (60 isolates, 69%) with the remainder approximately equally divided between *q*3 and *q*4. Norwegian isolates were predominantly assigned to either *q*3 or *q*4 populations, while the Australian isolates were all associated with cluster *q3*. Overall this suggests a shared ancestry for the English and Scottish isolates through *q1* and *q4*, while the Norwegian isolates appear to share ancestry with England, Scotland, and Australia via *q3* and *q4* (Figure 2B).

Principal component analysis using the microsatellite repeat size data separated the isolates into broad clusters representing country of origin (Figure 3C). However, the extensive diversity captured in the English isolates led to a spread of these isolates across the first component space, overlapping with the Australian, Scottish, and Norwegian isolates and the second component failed to resolve the different populations into clear clusters. When the binary matrix values of the microsatellite data were used, the number of variables increased from 8 to 122 thereby enhancing the discriminative power of the principal component analysis. This resulted in the isolates being resolved into clear population clusters, with the English and Scottish isolates being grouped together, and the Norwegian and Australian isolates forming individual distinct clusters (Figure 3D), which confirmed the results of the STRUCTURE analysis.

### Molecular analysis of *Sclerotinia sclerotiorum* isolates using IGS sequencing

**Figure 4:**
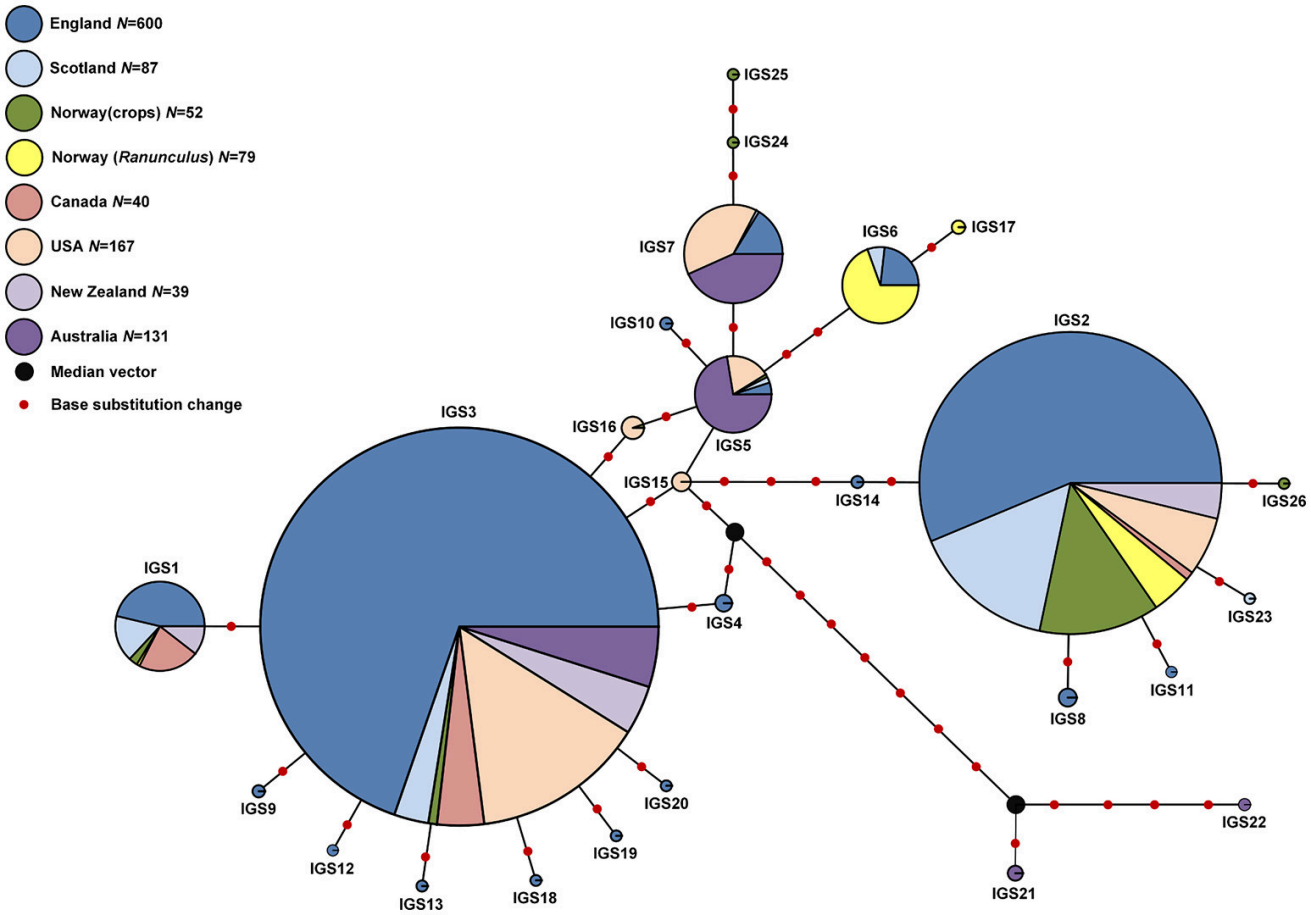
**Median joining networks showing phylogenetic relationships between IGS haplotypes for *S. sclerotiorum* isolates from Australia, England, Scotland, and Norway (this study) and Canada, USA, New Zealand and Lesser Celandine (*R. ficaria*) in Norway (Carbone and Kohn, 2001a)**. The size of each circle is proportional to the corresponding haplotype frequency. Branch lengths are proportional to the number of base substitution changes. A median vector represents a hypothesized haplotype required to connect existing haplotypes within the network.

In total, 26 IGS haplotypes were identified within the 870 *S. sclerotiorum* isolates (Table 8) from England (600 isolates), Scotland (87 isolates), Norway (52 isolates), and Australia (131 isolates). The most common haplotype was IGS3 (333 isolates), found in all three countries, closely followed by IGS2 (269 isolates) which was found in all countries except for Australia. In England, IGS3 (297 isolates) and IGS2 (179 isolates) were the most common haplotypes and were represented by isolates from every location and crop type as well as buttercup. In Scotland, the most prevalent haplotype was IGS2 (49 isolates) followed by IGS1 (16 isolates), both represented in all the buttercup and carrot populations genotyped. In Norway, the most prevalent haplotype was IGS2 (41 isolates) found in cabbage, camelina, lettuce, oilseed rape, and turnip rape from different locations. Conversely in Australia, the most common haplotype was IGS5 (58 isolates) followed by IGS7 (45 isolates) found in both lupin and oilseed rape across the majority of locations. A total of nine IGS haplotypes (IGS4, 8, 9, 10, 11, 12, 13, 20, 23) from England and Scotland were exclusively found in buttercup isolates. With these data, the haplotype network first published by Clarkson et al. (2013) was expanded considerably from 17 to 26 IGS haplotypes (Figure 4). The nine new haplotypes were from England (IGS18, Vowchurch oilseed rape 2009; IGS19, Sutton Bridge oilseed rape 2010; IGS20, Michaelchurch buttercup site 2, 2010), Australia (IGS21, oilseed rape different locations; IGS22, Mount Barker oilseed rape 2004), Scotland (IGS23, Dunfermline buttercup 2011), and Norway (IGS24, Buskerud lettuce 2013; IGS25, Nord-Trøndelag potato 2013; IGS26, Buskerud pumpkin 2013) (Genbank accession numbers KY798871-KY798879). The nearest neighbor statistic Snn values indicated that populations of *S. sclerotiorum* from different countries were all significantly differentiated from each other (Table 9).

**Table 8:** **IGS haplotype frequency and diversity for *S. sclerotiorum* isolates from England, Scotland, Norway, and Australia**.

**Haplotype**	**England**	**Scotland**	**Norway**	**Australia**	**Total**
IGS1	44	16	3	0	63
IGS2	179	49	41	0	269
IGS3	297	12	3	21	333
IGS4	15	0	0	0	15
IGS5	4	2	1	58	65
IGS6	19	6	0	0	25
IGS7	17	1	0	45	63
IGS8	10	0	0	0	10
IGS9	4	0	0	0	4
IGS10	1	0	0	0	1
IGS11	1	0	0	0	1
IGS12	2	0	0	0	2
IGS13	2	0	0	0	2
IGS14	2	0	0	0	2
IGS15	0	0	0	0	0
IGS16	0	0	1	0	1
IGS17	0	0	0	0	0
IGS18	1	0	0	0	1
IGS19	1	0	0	0	1
IGS20	1	0	0	0	1
IGS21	0	0	0	6	6
IGS22	0	0	0	1	1
IGS23	0	1	0	0	1
IGS24	0	0	1	0	1
IGS25	0	0	1	0	1
IGS26	0	0	1	0	1
Total isolates	600	87	52	131	870
No. haplotypes	17	7	8	5	
Haplotype diversity	0.659	0.632	0.377	0.663	

**Table 9:** **Nearest neighbor statistic (Snn values) for *S. sclerotiorum* populations from USA, Canada, New Zealand, Norway (Carbone and Kohn, 2001a), and England, Scotland, Australia, and Norway (Clarkson et al., 2013, this study)**.

	**USA^1^**	**Canada^1^**	**New Zealand^1^**	**Norway^1^**	**England^2^**	**Scotland^2^**	**Australia**	**Norway**
USA^1^								
Canada^1^	0.849^***^							
New Zealand^1^	0.795^***^	0.543^*^						
Norway^1^	0.927^***^	0.939^***^	0.869^***^					
England^2^	0.779^***^	0.898^***^	0.888^*^	0.916^***^				
Scotland^2^	0.783^***^	0.695^***^	0.626^**^	0.787^***^	0.796^***^			
Australia	0.742^***^	0.880^***^	0.876^***^	0.993^***^	0.936^***^	0.887^***^		
Norway	0.824^***^	0.811^***^	0.667^***^	0.821^***^	0.871^***^	0.565^**^	0.922^***^	

1 *S. sclerotiorum IGS sequences published by Carbone and Kohn (2001a). Isolates from cabbage, groundnut, oilseed rape, radish, tobacco (USA); oilseed rape (Canada); hemp, kiwi fruit (New Zealand), and lesser celendine (Norway)*.

2 Includes S. sclerotiorum IGS sequences published by Clarkson et al. (2013). Isolates from hosts in Table 1. Snn values significant at

*** P < 0.001,

** P < 0.01,

* *P < 0.05*.

## Discussion

There has been very little research concerning *S. subarctica* following the initial rDNA-based phylogeny of *Sclerotinia* species by Holst-Jensen et al. (1998) and the first report of the pathogen on crop plants in Alaska (Winton et al., 2006) where it was identified on potato, lettuce, cabbage, bean, and squash. This study shows for the first time a clear increase in the incidence of *S. subarctica* with increasing latitude from England, through to Scotland and Norway. In England, the pathogen appears to be insignificant compared to *S. sclerotiorum*, being identified in only one location (where originally first reported; Clarkson et al., 2010) on meadow buttercup despite widespread sampling. However, a much higher proportion of the *Sclerotinia* samples were identified as *S. subarctica* in Scotland (18%) and Norway (48%) with the latter very similar to the 46% reported in Alaska (Winton et al., 2006). Both Alaska and Norway occupy very similar latitude ranges. *S. subarctica* was also widely distributed across different crop plants and buttercup with new hosts reported here for the first time of carrot, celery root, Jerusalem artichoke, pea, and swede. This finding is therefore further evidence that *S. subarctica* has a broad host range similar to *S. sclerotiorum* or *S. minor*.

The reasons for the prevalence of *S. subarctica* in northern latitudes are unclear although it has been suggested that the larger sclerotia the pathogen generally produces may confer a survival advantage over *S. sclerotiorum* in harsh winters (Winton et al., 2006). Furthermore, we hypothesize that *S. subarctica* sclerotia may require an increased chilling requirement for rapid germination and apothecial production compared to *S. sclerotiorum* as observed in preliminary experiments (Warmington, 2014). If this is the case, then a lack of adequate cold temperature durations may therefore limit its reproductive ability, hence slowing down its spread further south. A requirement for cold “conditioning” for *S. sclerotiorum* sclerotia is well-documented for isolates from temperate regions (Phillips, 1987) although germination response to different temperature regimes varies between isolates from different geographic origins (Huang and Kozub, 1991). This, along with the extensive distribution of *S. sclerotiorum* (Anonymous, 2005), suggests adaptation to a much wider range of conditions for apothecial production than for *S. subarctica*.

This is the first study to extensively examine the population structure of *S. subarctica* and the finding that there are multiple clones (haplotypes) suggests a very similar reproductive strategy to *S. sclerotiorum* based on homothallic sexual reproduction through carpogenic germination of sclerotia. This is confirmed by observations of apothecial production by *S. subarctica* both in the laboratory (Warmington, 2014) and in the field (Winton et al., 2006). Furthermore, measures of linkage disequilibrium in the *S. subarctica* populations were significant which is again consistent with a selfing clonal population. This was also the case for the *S. subarctica* population from Alaska (Winton et al., 2007). This confirms the majority of studies with *S. sclerotiorum* where evidence of outcrossing is generally infrequent. However, more recent analyses using linkage disequilibrium decay with distance indicates that outcrossing can be much more common than is suggested solely by measurement of *I*_*A*_ (Attanayake et al., 2014).

Compared to the preliminary study in Alaska (Winton et al., 2007) where only four microsatellite haplotypes were identified within 41 *S. subarctica* isolates (10% of maximum), we identified a more diverse range of haplotypes in Scotland and Norway with 38 and 40 haplotypes found within 74 and 49 isolates respectively (51 and 82% of maximum). Furthermore, only 2–3 alleles were identified per locus in the Alaskan work compared to 5–10 alleles in this study. One possible explanation for this is that the Alaskan isolates were all collected from a confined area in the Matanuska Valley which was only developed for agriculture in the early 1900s. Therefore, there has been less opportunity for immigration of different *S. subarctica* haplotypes via crop plant or soil-based introductions compared to Scotland or Norway.

Another significant finding in our study is that a few *S. subarctica* haplotypes were found more frequently than the rest across multiple crops and locations. For instance, two microsatellite haplotypes which were the most prevalent in both Scotland and Norway were identified in 13 different locations in crops of carrot, lettuce, pea, potato, swede as well as buttercup. Scotland and Norway also shared a further six microsatellite haplotypes. This therefore follows the same pattern of distribution as observed for *S. sclerotiorum* both in this study and in previous research (Kohli et al., 1992; Kohn, 1995; Hambleton et al., 2002). The common haplotypes between Scotland and Norway suggests either a common origin and/or exchange of isolates and admixture between these two *S. subarctica* populations. This was supported by the STRUCTURE and PCA analyses where the majority of isolates from each country were assigned to the same clusters. Therefore, as suggested for *S. sclerotiorum* (Kohn, 1995; Hambleton et al., 2002), it seems likely that certain *S. subarctica* haplotypes persist following initial immigration due to the longevity of sclerotia in the soil with new haplotypes arising locally through mutation and infrequent outcrossing. Haplotypes present across multiple hosts including both crop plants and buttercup for both *S. subarctica* and *S. sclerotiorum* in this study confirms previous reports that there is no evidence for host specialization (Bolton et al., 2006) in either pathogen and that wild hosts can also potentially act as a source of inoculum on crop plants as well as enabling the pathogen to survive in the absence of a susceptible crop host.

In England however, *S. subarctica* was restricted to a single buttercup meadow in Herefordshire where repeated detection of the same microsatellite haplotypes each year indicated continual survival and cycling of the same pathogen isolates. Although repeat sampling of this meadow makes direct comparisons with the populations from Scotland and Norway problematic, none of the English *S. subarctica* microsatellite haplotypes were found in Scotland or Norway and they were also clearly assigned to a different population cluster in both the STRUCTURE and PCA analyses. The lack of evidence for admixture in the English isolates suggests that the same ancestral population has endured in isolation without any influx of additional genetic diversity resulting in fixation of alleles. This may have been caused by a founder effect following an initial introduction, with the initial population being a skewed sampling of the alleles from an overall larger population. Evidence for the isolation of the English *S. subarctica* population was further supported by highly significant differentiation from Scottish and Norwegian populations and as well as low genetic diversity with only five microsatellite haplotypes identified. A similar situation was described for an isolated *S. sclerotiorum* population on *R. ficaria* in Norway which was characterized by low diversity and apparent localized inbreeding (Kohn, 1995). In addition, despite the availability of susceptible hosts in the area *S. subarctica* was not identified elsewhere locally. For instance, no *S. subarctica* was identified in an adjacent buttercup meadow (Michaelchurch Escley2, Herefordshire) approx. 100 m away (but separated by a road and hedges), although affected by *S. sclerotiorum*. Similarly, *S. subarctica* was not detected in two different oilseed rape fields 6 km away (Vowchurch 2009 and 2010), where *S. sclerotiorum* was again identified. This could suggest a limited ability of *S. subarctica* to spread, despite its potential ability to produce airborne ascospores via apothecia (Winton et al., 2006; Warmington, 2014). However, studies with *S. sclerotiorum* have shown that the majority of ascospores may only travel 40–60 m while long distance dispersal depends on wind speed and direction (Qandah and Del Rio Mendoza, 2012).

Populations of *S. sclerotiorum* from England, Scotland, Norway, and Australia were also analyzed in this study and in contrast to our previous work which only examined population structure at regional level in England (Clarkson et al., 2013), these additional isolates allowed us to examine populations at a different spatial (country) scale. Although clonal diversity measures were significantly lower for Australia compared to the other countries, especially for *Hc*, these estimates were comparable to other studies for *S. sclerotiorum* using microsatellites (e.g., Attanayake et al., 2013; Aldrich-Wolfe et al., 2015). The 15 shared microsatellite haplotypes between England and Scotland, assignment of the majority of isolates to two common clusters in the STRUCTURE analysis and a common cluster in the PCA analysis indicated admixture of these populations from a common origin which would be expected in adjacent countries. In contrast, the Australian *S. sclerotiorum* isolates were quite different from English/Scottish isolates as they were all assigned to different clusters in the STRUCTURE/PCA analyses suggesting a different ancestry which most likely reflects their geographic separation. However, despite the *S. sclerotiorum* isolates from Norway being separated from English and Scottish isolates in the PCA analysis, some appeared to potentially share some ancestry with both England/Scotland and Australia in the STRUCTURE analysis. Furthermore, in contrast to *S. subarctica*, there were no shared haplotypes between *S. sclerotiorum* populations in Norway and Scotland. This would therefore suggest a different pattern of initial distribution of *S. sclerotiorum* compared to *S. subarctica*.

The IGS sequence data for *S. sclerotiorum* provided a different level of phylogenetic resolution than the microsatellite data and a further nine new IGS haplotypes were identified, adding to the original 17 described by Clarkson et al. (2013), for *S. sclerotiorum* populations from the UK and previously published sequence data for populations from Canada, New Zealand, Norway, and the USA (Carbone and Kohn, 2001a). However, the new haplotypes were at low frequency while haplotype IGS 3, which was previously commonly found within *S. sclerotiorum* populations from all the above countries, was also identified in the Norwegian and Australian populations. The next most common haplotype IGS 2 was found in all countries except Australia. IGS sequencing therefore allows effective comparisons between different *S. sclerotiorum* populations globally and the frequency and relationship of the IGS haplotypes seen in the phylogenetic network continues to suggest the wide distribution of a small number of common haplotypes, with lower frequency haplotypes often emerging from these at the local scale as suggested by Clarkson et al. (2013).

Overall therefore, *S. subarctica* populations from Scotland and Norway appear admixed, with a common origin and shared microsatellite haplotypes. The lower incidence of *S. subarctica* in Scotland than in Norway and the rare occurrence of the pathogen in England may suggest a possible north-south migration and that the UK is at the limit of the pathogen's southerly distribution. However, further data and analysis would be required to test this theory. In contrast, for *S. sclerotiorum*, English and Scottish populations were similar, with shared microsatellite haplotypes and a common origin for many of the isolates. The Norwegian population showed only partial evidence of a common ancestry, and as for the Australian population, was clearly distinguished by PCA analysis. This therefore suggests limited admixture and geographic isolation between *S. sclerotiorum* populations from the UK, Norway, and Australia.

## Author contributions

JC conceived and obtained funding for the UK work, obtained isolates from diseased plants, carried out microsatellite genotyping and IGS sequencing of *S. sclerotiorum* and *S. subarctica* isolates and wrote paper. RW obtained isolates from diseased plants, carried out microsatellite genotyping and IGS sequencing of *S. sclerotiorum* and *S. subarctica* isolates and co-wrote paper. PW carried out Structure and PCA analyses of microsatellite data and edited paper. MD obtained isolates from diseased plants and carried out IGS genotyping of Australian *S. sclerotiorum* isolates. MB and GB obtained isolates from diseased plants and edited paper. BN conceived and obtained funding for the Norway work, obtained isolates from diseased plants and edited paper.

### Conflict of interest statement

The authors declare that the research was conducted in the absence of any commercial or financial relationships that could be construed as a potential conflict of interest.
